# The functional magnetic resonance imaging (fMRI) procedure as experienced by healthy participants and stroke patients – A pilot study

**DOI:** 10.1186/1471-2342-9-14

**Published:** 2009-07-31

**Authors:** André J Szameitat, Shan Shen, Annette Sterr

**Affiliations:** 1 Department of Psychology, University of Surrey, Guildford, UK; 2 Department of Psychology, Ludwig Maximilians University, Munich, Germany

## Abstract

**Background:**

An important aspect in functional imaging research employing magnetic resonance imaging (MRI) is how participants perceive the MRI scanning itself. For instance, the knowledge of how (un)comfortable MRI scanning is perceived may help institutional review boards (IRBs) or ethics committees to decide on the approval of a study, or researchers to design their experiments.

**Methods:**

We provide empirical data from our lab gained from 70 neurologically healthy mainly student subjects and from 22 mainly elderly patients suffering from motor deficits after brain damage. All participants took part in various basic research fMRI studies using a 3T MRI scanner. Directly after the scanning, all participants completed a questionnaire assessing their experience with the fMRI procedure.

**Results:**

87.2% of the healthy subjects and 77.3% of the patients rated the MRI procedure as acceptable to comfortable. In healthy subjects, males found the procedure more comfortable, while the opposite was true for patients. 12.1% of healthy subjects considered scanning durations between 30 and 60 min as too long, while no patient considered their 30 min scanning interval as too long. 93.4% of the healthy subjects would like to participate in an fMRI study again, with a significantly lower rate for the subjects who considered the scanning as too long. Further factors, such as inclusion of a diffusion tensor imaging (DTI) scan, age, and study duration had no effect on the questionnaire responses. Of the few negative comments, the main issues were noise, the restriction to keep still for the whole time, and occasional feelings of dizziness.

**Conclusion:**

MRI scanning in the basic research setting is an acceptable procedure for elderly and patient participants as well as young healthy subjects.

## Background

There has been a boom in human neuroscience studies using magnetic resonance imaging (MRI), and a number of sites introduce MRI as a new technique. Here, researchers as well as administrative personnel, such as members of ethics committees, enter a new domain in which they often have little background knowledge. Of particular importance in this context are concerns about the well being of the participants tested in the MRI machine [1-13]. The test situation may not be the most pleasant given the narrow diameter of the scanner tube, the noise of the gradient coils, and the fact that participants are asked to lie still for an extended period of time. MRI machines are typically approved by local governmental health agencies for clinical use (e.g. by the Medicines and Healthcare products Regulatory Agency (MHRA) in the UK). But it is also important to establish how comfortable experimental scanning procedures are when basic research protocols are used. Knowledge on participants' experience of the MRI scanning procedure may inform decisions of ethical committees as well as of researchers when planning an experiment (e.g., duration of a scanning session, the likelihood of movement artifacts [8]).

While a number of studies investigated the perception of MRI scanning in the clinical setting, there is, to our knowledge, no study which assessed the perception of MRI scanning under the particular circumstances of basic imaging research (see Wollman et al. [4] for an exception in elderly participants). As elaborated in the Discussion section, there are a number of differences between clinical and research scans, such as selection of patients/participants, noise of the scanning environment, or scan duration. We therefore conducted a pilot study and subjected participants undergoing a research scanning procedure in our laboratory to a post-scan questionnaire, which was designed to assess the MRI experience. The scanning procedure consisted of functional EPI scans, an anatomical magnetization prepared rapid gradient echo (MPRAGE) scan, and, for roughly half of the participants, a diffusion tensor imaging (DTI) scan. We report the results of two independent and unrelated populations as investigated in our laboratory in the context of different ongoing fMRI studies, i.e. healthy subjects and patients with sustained motor deficits and reduced mobility following brain damage.

## Methods

### Sample

70 neurologically healthy subjects (39 female, 31 male) completed the questionnaire subsequent to the MRI scan. Participant's age ranged from 17 to 60 years, with a mean age of 26 years (standard deviation 10.6 years). Although we do not have data on this, the authors (who conducted all studies personally) estimate that approximately one third of the subjects participated in an fMRI study before. Subjects were paid between €10 and €15 for participation.

In addition, 22 clinical patients (5 female, 17 male; aged 27–69 years, mean 54.1 years, standard deviation 12 years) suffering from a chronic hemiplagia after stroke (chronicity > 1 year) completed the questionnaire. These patients had unsystematically mixed motor impairments of either the left or right side. Except for a few exceptions patients had no obvious cognitive or speech/language impairments. Approximately half of them had a clinical MRI scan before. These 22 patients were derived from a sample of 42 patients receiving motor rehabilitation in our laboratory. 20 of the 42 patients did not undergo MRI mainly due to ferromagnetic materials in their body or due to other conditions (e.g. diabetes or heart diseases) which we took as exclusion criteria in the context of basic research. All patients were scanned twice within a 2–3 week interval, and the questionnaires were presented only at the first scanning session. Participation in the MRI scanning was voluntary.

In the following, with the term "participants" we refer to the combined sample of healthy subjects and patients. All participants were recruited for research studies not related to the present questionnaire study.

The present questionnaire study and the experiments were approved by the ethics committee of the University of Surrey (UK), and all participants gave written informed consent prior to participation.

### The questionnaire

The questionnaire was initially developed as quality assurance instrument for internal use. The questionnaire consisted of 4 questions regarding the MRI scanning procedure and 9 questions (not reported) regarding the experiment itself. The questions of interest were (1) How was the scanning procedure? [answer via 7 point scale with the endpoints 1/very comfortable and 7/very uncomfortable; option for additional comments], (2) Do you think you felt something "strange" caused by the MRI scanner (e.g. dizzy)? [yes/no; If yes, please describe], (3) Was the scanning too long? [yes/no], the healthy subjects in addition were asked the question (4) Would you like to participate in an MRI study again? [yes/no].

The questionnaire was generally filled out by the participants themselves after the MRI scan. For patients with writing difficulties the experimenter filled out the questionnaire. We did not always check immediately whether the questionnaire was filled out completely so that participants may have left a question unanswered for unknown reasons (e.g. missed, refused to answer).

### Studies

Generally, our lab is focused on research of the motor system. The healthy subjects were tested in the context of six different studies (Table 1). In three of these studies subjects had to perform isometric force tasks with their dominant hand, and in the other three tasks they had to perform overt movements in combination with motor imagery, or sole motor imagery. Some of the studies have been published previously [14-17]. The patients had to perform an isometric force task with their affected hand.

**Table 1:** Sample description.

Study	N (fem)	Age ± s.d.	Duration	Paradigm
1	10 (8)	22.5 ± 2.5	53 (47–55)	press handle with varying pace and force

2	19 (11)	25.0 ± 6.6	40 (37–45)	motor imagery, passive movement, movement observation

3	15 (9)	21.0 ± 3.6	46 (40–48)	motor imagery

4	10 (6)	29.3 ± 9.3	20 (19–27)	motor imagery

5	11 (2)	20.6 ± 3.0	40 (33–41)	squeezing a ball with both hands

6	5 (3)	57.4 ± 2.7	29	press handle with varying force

*total*	*70 (39)*	*26 ± 10.6*	*39.5 ± 10.5*	

**Patients**				

Study	N (fem)	Age ± s.d.	Duration	Paradigm

1	22 (5)	54.1 ± 12.0	21	press handle with varying force

Number of participants (N) and, among these, number of females (fem) is given. Age and standard deviation (s.d.) in years. Duration is the actual average duration, in brackets the duration range is given (without DTI – with DTI).

All participants underwent an anatomical scan of 5 minutes. If the study consisted of two or more functional runs, the anatomical scan was always performed between the first and second functional run, otherwise it was performed after the functional run. If a DTI scan (lasting 8 min) was performed, it was always the last scan of the session.

The study with patients consisted of two functional runs, each lasting 8 min, resulting in a total scanning duration of 21 min. Regarding the healthy subjects, five of them underwent the same protocol as the patients, except that they had in addition a DTI scan. The first study (10 subjects) consisted of two functional runs lasting 24 min and 18 min, respectively (total duration 47–55 min depending on whether DTI was performed). The second study (19 subjects) consisted of two functional runs, each lasting 16 min (total duration 37–45 min). The third study (15 subjects) also consisted of two functional runs lasting 18 and 17 min, respectively (total duration 40–48 min). The fourth study (10 subjects) consisted of a single functional run lasting 14 min (total duration 19–27 min). The fifth study (11 subjects) consisted of two functional runs, each lasting 14 min (total duration 33–41 min). The average scanning time was 39.5 min (standard deviation 10.51 min).

The given times are the pure scanning times, without short gaps between the scans, setting up the scanner, or re-starting the scanner due to problems. On average, each person stayed approx. 5–10 min longer in the scanner bore than specified above, resulting in estimated total durations between 24 min and 65 min.

### MRI procedure

**Figure 1:**
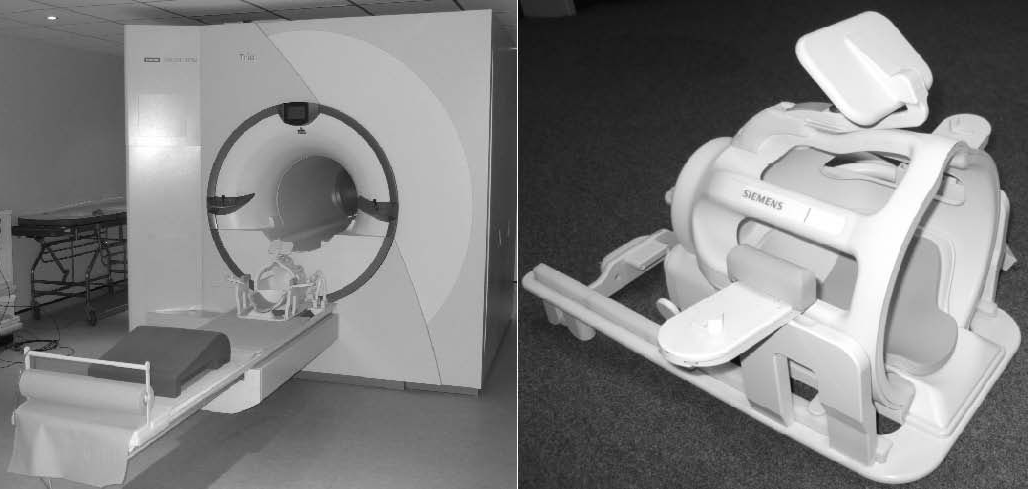
**MRI equipment**. Siemens Trio 3 Tesla MRI scanner (left) and the used array head coil with attached mirror to enable visual stimulus presentation (right). During scanning two additional foam cushions, one on each side, were placed between the fasteners and the participant's head to further shield the noise and reduce head motion.

Imaging was carried out using a 3 Tesla scanner (Trio, Siemens, Erlangen, Germany) equipped with an array head coil (Figure 1). The bore had a diameter of 60 cm (length 180 cm), and participants were inside the bore approximately up to their abdomen/hip region. Participants were supine on the scanner bed, and cushions were used to reduce head motion. A leg rest was placed under the thigh and calves which makes lying on the back more comfortable, especially for the back. If required, pads were put under the upper arm to support holding a response device. No further devices such as straps or vacuum cushions were used. Participants were given earplugs to lower the noise level (up to approx. 100 dBA SPL inside the bore without protection). A mirror, attached on the top of the headcoil enabled the participants to view the screen at the top end of the scanner bore. 36 axial slices (192 × 192 mm field of view (FOV), 64 × 64 matrix, 4 mm thickness, no gap, interleaved slice acquisition) were acquired using an EPI sequence (repetition time (TR) 2 s, echo time (TE) 30 ms, 90° flip angle). In each study, a high-resolution whole brain image was acquired from each participant using a T1-weighted MPRAGE sequence (1 × 1 × 1 mm voxel size). At the end of the scanning session, for 42 of the 70 (60%) healthy subjects a DTI scan lasting 8 min was conducted.

### Statistics

The binary questionnaire items (with a "yes/no" answer) were treated as nominal data and analyzed using Chi-square (χ^2^) tests. The response to the question "How was the scanning procedure?" was given on a 7-point scale. We treated this as interval scale and used parametric tests (independent samples t-tests, one-sample t-tests, Analyses of Variance (ANOVAs)). We validated the results by calculating non-parametric analyses (Mann-Whitney-U tests, Wilcoxon signed rank tests, Kruskal-Wallis test). Although parametric and non-parametric tests always revealed the same pattern of results we present both results where appropriate.

For the correlation of two interval scale items (e.g. age and the item "how was the scanning procedure") we used Pearson's correlation (validated by non-parametric Spearman's rho correlation). Relations between nominal-scale items and interval-/ordinal-scale items were assessed using Spearman's rho correlation.

## Results

### Response rates

Each question was answered by more than 94% of the subjects and patients, with the only exception of the question whether they would like to participate again, which was answered by 87.1% of the healthy subjects (this question was not presented to the patients).

### Questionnaire responses

Questionnaire responses are summarised in Table 2. Each questionnaire item is presented in detail below.

**Table 2:** Summary of the questionnaire results.

	Question 1 „comfortable"		Question 2 „strange"		Question 3 „too long"		Question 4 „do it again"
	healthy	patients	healthy	patients	healthy	patients	healthy

number of respondents	68	22	68	21	66	22	61

means or percentages	3.03*** ^1^	2.73** ^1^	33.8%*** ^2^	28.6%*** ^2^	12.1%*** ^2^	0%	93.4%*** ^2^

healthy vs. patients	ns		ns		ns		N/A

*effect of*							

Question 2 „strange"	ns	ns	--	--	*** ^3^	ns	** ^3^

Question 3 „too long"	ns	ns	*** ^3^	ns	--	--	*** ^4^

Question 4 „do it again"	ns	ns	** ^3^	ns	*** ^4^	N/A	--

having a DTI scan	ns	N/A	ns	N/A	ns	N/A	ns

study^5^	ns	N/A	ns	N/A	ns	N/A	ns

study duration^6^	ns	N/A	ns	N/A	ns	N/A	ns

gender	* ^7^	* ^7^	ns	ns^8^	ns	ns	ns

males	2.63	3.12	45.2%	25%	10%	0%	88.9%

females	3.34	1.40	24.3%	40%	13.9%	0%	97.1%

Abbreviations: * p < .05; ** p < .01; *** p < .001; ns not significant; N/A not applicable

Footnotes: ^1 ^one-sample t-test versus mean of 4; ^2 ^χ^2 ^test versus 0% (Questions 2 and 3) or 100% (Question 4); ^3 ^people noting something strange more often found the session too long and were less willing to participate again; ^4 ^people finding session too long are less willing to participate again; ^5 ^tested by one-way ANOVA (Question 1) or χ^2 ^test (Questions 2–4); ^6 ^tested by Pearson's r (Question 1) or by Spearman's rho (Questions 2–4); ^7 ^significant interaction (p = .002) between gender and participant group as assessed by 2 × 2 ANOVA; ^8 ^trend with p < .07. For further statistical details see main text.

Healthy refers to the sample of healthy subjects and patients to the sample of stroke patients. For question 1, mean ratings of the 7-point scale are given. For questions 2 to 4 the data refer to the proportion of "yes" answers (i.e. feeling something strange, considering the scanning as too long, and would like to participate again). The rows "effect of" indicate whether the named factors significantly affected the answer to the questionnaire items. For instance, Question 1 was answered the same, irrespective of the answer on Question 2, while Question 3 differed in healthy controls depending on the answer on Question 2. Note that the number of males and females is not balanced. Question 4 was not presented to the patients.

#### 1) How was the scanning procedure?

**Figure 2:**
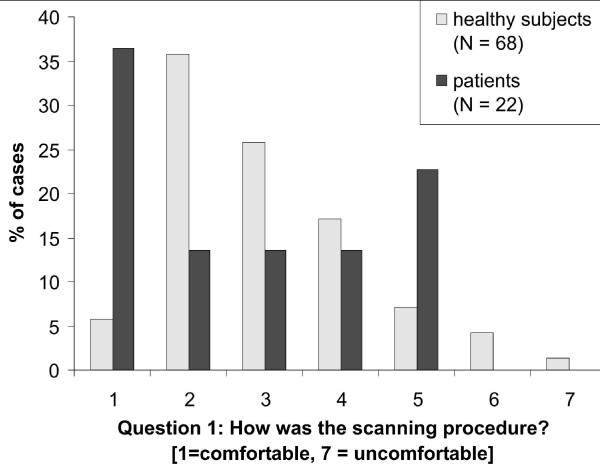
**Histogram of the responses for the question "How was the scanning procedure?"**. Participants responded on a scale from 1 (very comfortable) to 7 (very uncomfortable). Y-axis depicts the proportion of cases who chose a given response category. Healthy subjects as well as patients rated the procedure on average as being comfortable (ps < .01 when compared to the middle category of 4). Mean ratings of healthy subjects and patients did not differ significantly.

68 healthy subjects responded on a scale from 1(very comfortable) to 7 (very uncomfortable), with a mean of 3.03 (s.d. 1.31) and a median of 3 (range 1–7))(Figure 2, light grey bars). This data showed a significant positive trend in evaluating how comfortable the scanning procedure was (one-sample t-test versus 4 (center of the rating scale): t(67) = 6.08; p < .001; Wilcoxon signed rank test versus 4: Z = 4.776, p < .001). Ten subjects commented with "noisy", "tiring", "slightly claustrophobic", "nerve racking", "head and neck were sore", "inability to scratch itches", "back hurt" (two subjects), and "headphones uncomfortable" (two subjects).

22 patients responded (Figure 2, dark grey bars) with a mean of 2.73 (s.d. 1.64) and a median of 2.5 (range 1–5). Similar to healthy participants, patients showed a significant positive trend in evaluating how comfortable the scanning procedure was (one-sample t-test versus 4 (center of the rating scale): t(21) = 3.644; p = .002; Wilcoxon signed rank test versus 4: Z = 2.97, p = .003). The only three comments made were "noise", "too noisy", and "I'm big to fit in".

Although the exact distribution of rating scores differed between healthy subjects and patients (Pearson χ^2 ^= 20.605, df = 6, p = .002), the mean rating scores did not differ between the groups (healthy participants = 3.03; patients = 2.73; t(88) = .880; p = .381; Mann-Whitney-U test: Z = .983; p = .326).

The rating on comfort was largely independent of feeling something strange (independent samples t-tests; patients: t(19) = .994, p = .333; healthy subjects: t(65) = 1.25, p = .216), future participation (healthy subjects: t(59) = .027, p = .978; item was not presented to patients), and whether subjects considered the scanning session as too long (healthy subjects: t(64) = 1.393, p = .168; no patient considered the session as too long).

#### 2) Do you think you felt something "strange" caused by the MRI scanner (e.g. dizzy)?

68 healthy subjects answered this question with 23 (33.8%; χ^2^-test for the null hypothesis that no subject feels something strange: χ^2 ^= 740.02; df = 1; p < 0.001) reporting having felt something strange. In more detail, 7 subjects reported tiredness, 5 dizziness during the scan, and 2 feeling slightly discoordinated and disoriented. One subject reported a feeling of panic caused by the enclosed space in the MRI bore and the noise of the anatomical MPRAGE scan. Further comments were "seeing stars against the [bright] white screen" and "feeling wobbly when standing up after the end of the MRI scan". Two subjects reported slight nausea after the DTI scan, during which the scanner bed shakes noticeably. Further various comments are discussed in the Discussion section.

21 patients answered this item and 6 of them (28.6%; χ^2 ^= 161.25; df = 1; p < 0.001) noted something strange. Four patients reported slight dizziness, one tiredness, and one a "little tingle in right leg".

The percentage of healthy controls (33.8%) and patients (28.6%) noting something strange did not differ significantly (Pearson χ^2 ^= .201; df = 1; p = .654).

In healthy subjects the perception of something strange was related to considering the scanning session as too long. In more detail, 7 of 22 subjects (31.8%) noting something strange considered the session as too long, while only 1 of the 43 subjects (2.3%) noting nothing strange considered the session as too long (Pearson χ^2 ^= 11.729, df = 1, p = .001). No patient considered the session as too long.

A comparable pattern emerged for the relation between feeling something strange and the question whether they would like to participate again (not presented to patients). All 39 subjects who didn't note anything strange would have participanted again. Out of the 21 participant who noted something strange, only 17 (81%) considered future participation (Pearson χ^2 ^= 7.959, df = 1, p = .005).

#### 3) Was the scanning too long?

66 healthy subjects answered this question and 8 of them (12.1%; χ^2 ^= 82.454; df = 1; p < .001) considered the scanning session as too long. 22 patients answered this question and none considered the scanning session as too long.

The rate of healthy subjects (12.1%) and patients (0%) considering the session as too long was not significantly different, potentially due to a lack of power (Pearson χ^2 ^= 2.933; df = 1; p = .087).

#### 4) Would you like to participate in an MRI study again?

61 healthy subjects answered this item and 57 of them (93.4%) considered future participation, but 4 subjects did not 4 (6.6%; χ^2 ^= 19.03; df = 1; p < .001). This item was not presented to patients. However, all patients visited the MRI unit twice within a 3-week period and no participant cancelled the second scan.

In healthy subjects future participation depended on the length of the scanning session. In detail, 8 subjects considered the session too long and only 5 of them (62.5%) would have liked to participate again. 51 subjects took no issue with scanning length and most of them (50; 98%) considered future participations (Pearson χ^2 ^= 13.82, df = 1, p < .001).

### The effect of an additional DTI scan

The DTI scan had no effect on the perception of comfort (two-sample t-test: t(66) = .033; p = .974; Mann-Whitney U test: Z = .162; p = .871) and was unrelated to the perception of "something strange". The latter was reported in 31.7% (13 of 41) of subjects undergoing DTI and 37% (10/27) of subjects not undergoing DTI scan (Pearson χ^2 ^= .207; df = 1; p = .649). Further on, the experiment was considered too long by 10.7% (3/28) of subjects undergoing DTI and 13.2% (5/38) of volunteers not undergoing DTI. This difference was insignificant (Pearson χ^2 ^= .090; df = 1; p = .764). Finally, 94% (34/36) of subjects receiving DTI would like to participate again, while 92% (23/25) of subjects without DTI considered future participation. Again this difference was insignificant (Pearson χ^2 ^= .144; df = 1; p = .704).

### The effect of study and study duration

To generally assess whether the study affected the questionnaire results, we first calculated a one-way ANOVA with study as between-group factor. This analysis was performed for healthy controls only, since the patients participated only in one study paradigm. The analysis showed that study had no effect on perveived comfort (F(5, 62) = .447; p = .814; Kruskal-Wallis nonparametric ANOVA χ^2 ^= 1.708; df = 5; p = .888). In addition, the factor study had no effect on the perception of something strange (Pearson χ^2 ^= 5.026; df = 5; p = .413), judgment on study length (Pearson χ^2 ^= 1.860; df = 5; p = .868), or future participation (Pearson χ^2 ^= 5.146; df = 4; p = .273).

Although within a study always the same EPI scans were performed (resulting in a constant scanning time), only some subjects underwent a DTI scan, which resulted in different total scanning times even within a study. To account for this variability, the above analysis was repeated with the factor *study duration *instead of *study*, which essentially revealed the same effect pattern. Perception of comfort and scan duration were not correlated (Pearson's r = -0.095; p = .442; Spearman's rho = -0.067; p = .559). Furthermore, study duration had no effect on the perception of something strange (Spearman's rho = .075; p = .543), the judgment on study length (Spearman's rho = .097; p = .438), or future participation (Spearman's rho = -0.046; p = .727).

### The effect of gender

Gender affected the results significantly. Female participants in the healthy group (N = 38; mean 3.34) rated the scanning procedure as significantly less comfortable than males (N = 30; mean 2.63; two-sample independent t-test: t(66) = 2.274; p = .026; Mann-Whitney U test: Z = 2.112; p = .035). For the patients a reversed pattern emerged, with females (N = 5; mean 1.40) rating the procedure as more comfortable than males (N = 17; mean = 3.12; independent t-test: t(20) = 2.252; p = .036; Mann-Whitney U test: Z = 2.148; p = .039). This interaction was significant, as tested by a 2 × 2 factorial ANOVA with group and gender as between subject factors (interaction between group and gender: F(1, 86) = 10.426; p = .002; main effects n.s.). Given that the groups differed in their mean age, the ANOVA was repeated with age as co-variate of no interest. This analysis yielded similar results (interaction between group and gender: F(1, 85) = 10.438; p = .002; effect of age: F(1, 85) = 2.141; p = .147; main effects n.s.)

Furthermore, healthy males tended to note more often something strange than females (45.2% males; 24.3% females; Pearson χ^2 ^= 3.272; df = 1; p = .07). However, this trend was absent in patients (4 of 12 males (25%); 2 of 3 females (40%); Pearson χ^2 ^= .420; df = 1; p = .517).

With regards to length of scan no gender differences were found between groups (Pearson χ^2 ^= .232; df = 1; p = .630).

Finally, 2.9% of females and 11.1% of males stated they would not like to participate again, which did not differ significantly from each other (Pearson χ^2 ^= 1.639; df = 1; p = .2). This question was not presented to the patients.

### The effect of age

To assess the effect of age we first calculated the correlation between age and how comfortable the procedure was rated. No significant correlations emerged (healthy subjects: Pearson's r = .161, p = .190; Spearman's rho = .192, p = .117; patients: Pearson's r = .164, p = .467; Spearman's rho = .115, p = .612; healthy subjects and patients pooled (N = 90): Pearson's r = .034, p = .753; Spearman's rho = .058, p = .587).

Age also had no effect on the perception of something strange (pooled: Spearman's rho = -0.098, p = .362; patients only: Spearman's rho = -0.139, p = .547; healthy subjects only: Spearman's rho = -0.086, p = .486), on judgment of scanning length (pooled: Spearman's rho = -0.123, p = .253; healthy subjects only: Spearman's rho = -0.012, p = .922; no patient considered the session as too long), and future participation (Spearman's rho = -0.051, p = .696).

## Discussion

The present report aimed at providing information about how two independent populations, one consisting mainly of neurologically healthy students (N = 70) and one of elderly stroke survivors (N = 22), perceive the functional MRI procedure. For this, we presented a questionnaire asking for their opinion on the MRI scan directly after the scanning had finished. The results showed that the majority of healthy subjects as well as patients consider MRI scanning as a comfortable procedure, and that virtually all subjects would like to participate in a subsequent scanning. As negatives, mainly the scanning noise, the need to lie still and not move the head, and occasional feelings of dizziness were mentioned.

### How convenient is a basic research scan?

Previous studies primarily assessed the tolerance but not the comfort of the MRI procedure. In particular, anxiety and the occurrence of claustrophobia during MRI scanning has been in the focus of previous research, probably because claustrophobia is the most severe problem and typically results in scan abortion [3,12,18-23]. This is perfectly reasonable for the clinical setting in which MRI scans serve important diagnostic purposes and for which the potential health benefits outweigh patient discomfort. However, in the basic research setting researchers and ethical review boards typically consider only moderate levels of discomfort as acceptable. Accordingly, we designed our questionnaire to assess the perception of MRI scanning not on a coarse level (scan was possible vs. had to be aborted) but on a more subtle level of perceived comfort.

Our data show that healthy subjects and patients generally found the scanning procedure comfortable. 67% of the healthy group and 63% of the patients rated the procedure positively (i.e. between 1 and 3 on a 1–7 scale (central item is 4)), and only 13% of healthy subjects and 23% of patients rated the procedure negatively (i.e. between 5 and 7). Extreme negative ratings were rare, as only 1 out of 68 healthy controls rated the procedure as 7 (very uncomfortable), and 2 rated it as 6. No patient rated the procedure as 6 or 7. This demonstrates that the large majority of participants is fine with the procedure and that only very few participants consider the MRI procedure to be very uncomfortable.

These findings are in line with the few previous studies investigating the comfort of the MRI procedure. When the rating scales given to the participants in the different studies are transformed to a universal scale ranging from 0 [uncomfortable] – 100 [comfortable] by the formula (100/(number of choices on response scale - 1) × (mean rating - 1)), we observed in the present study a mean rating of 66.2 for the healthy subjects and 71.2 for the patients. Wollman et al. [4] also asked how comfortable the overall experience of a research scan was and reported a transformed mean rating of 77.5 for a sample of participants older than 72 years. Sparrow et al. [2] asked patients after a clinical scan for the comfort of the scan. Although the scan involved injection of a contrast agent, the transformed mean rating still was 63.6, i.e. only slightly below the one observed in the present study. Dantendorfer et al. [8] used only a very broad scale which unfortunately cannot be transformed in a clinical study comparing a 0.5T and a 1.5T scanner. He reported that 80% (1.5T) – 88% (0.5T) of the patients found the MRI procedure easy to tolerate, 18% (1.5T) – 11% (0.5T) found it unpleasant, and only 2.2% (1.5T) – 0.7% (0.5T) found it hardly bearable. Although the rating scales are not directly comparable, we think that the findings of Dantendorfer et al. [8] are in general agreement with our findings. Taken together, the present finding that participants regard MRI research scans as comfortable is in line with previous studies investigating the research setting in the elderly [4] as well as studies investigating the clinical setting [2,8].

A further finding supporting this conclusion regards the wish to participate again in an fMRI study. In the present study 93.4% of the healthy subjects would like to participate in an fMRI study again. In more detail, 98% of those participants who did not consider the session as too long would like to participate again, but only 62.5% of the subjects who considered the session as too long would do so. Thus, too long scanning sessions may considerably reduce the willingness to participate again. However, it should be noted that this conclusion is based on rather small absolute numbers, since only 8 subjects in total considered the scanning session as too long.

Our findings are in agreement with Wollman et al. [4] who reported that 100% of their (elderly) subjects would undergo the MRI procedure again (note the active wish to participate again in our study, as compared to the more passive agreement that they would do it again in Wollman et al. and MacKenzie et al.). MacKenzie et al. [6] reported for a clinical context a willingness to return of only 64%, with an additional 24% returning only if absolutely necessary. The reason for the lower rate is not clear, but may be found in longer scanning durations (up to 95 min in MacKenzie et al.), differences in the procedure (e.g. in MacKenzie et al. scans of many different body parts were analyzed), or differences between clinical and research scans (see next section below).

### MRI in the clinical and in the basic research setting

Clinical and research scans differ in some important aspects so that the results gained from one may not hold for the other. First, MRI research settings may impose greater discomfort than clinical scans. For instance, MRI research employs different imaging parameters. Critically, the most frequently employed sequence in research, i.e. echoplanar imaging (EPI), is characterized by a very high volume hammering gradient noise. A further difference is that clinical scans usually require the patient to lie passively in the MRI scanner, while the experimental tasks in research scans can be quite demanding (mentally as well as physically as for instance in the case of the patients who were required to press a handle with the arm affected by a stroke). Interestingly, these differences do not seem to affect the perceived comfort, since the present study and Wollman et al. [4] found comparable ratings of comfort as compared to clinical studies [2,8].

Furthermore, research scans tend to take longer than clinical scans. While clinical scans take typically less than 30 minutes, research scans frequently take 45 – 75 minutes, with durations up to 120 minutes. It appears plausible to assume that longer scanning duration increases the experienced discomfort, at least beyond some point. However, only few healthy subjects (8 of 66; i.e. 12.1%) considered the scanning session as too long. Notably, this was neither correlated to the actual study duration nor related to the comfort ratings. This suggests that healthy participants seem to be fine with scanning sessions of at least up to 1 h in total.

On the other hand, one may hypothesize that clinical scans are perceived as less comfortable than research scans. For example, anxiety levels in clinical scans are high, with up to 37% of patients reporting moderate to high levels of anxiety [7,10,11], up to 6.5% of aborted scans [24,25], and up to 14.2% of patients needing sedation to tolerate MRI [26].

These scans are prescribed and usually conducted for diagnostic purposes. Patients may therefore undergo this procedure despite their anxiety. The prospect of the diagnosis may further aggravate anxiety levels in general and make participants more susceptible to the feeling of anxiety in the scanner setting [5,6,11]. In contrast to the clinical environment, participation in MRI scanning is voluntary, and one would therefore assume that persons who worry about the scanning experience would simply not volunteer to participate in those studies [21]. In other words, for research-based scanning a self-selection bias automatically leads to a cohort of volunteers who are likely to be comfortable with the scanning procedure. However, the present evidence does not directly support this conclusion. All previous studies investigating the comfort of the MRI procedure showed that participants and patients generally perceive the procedure as comfortable, irrespective of age or the use of contrast agents. Whether there are truly no differences between clinical and research scans or whether larger samples and improved questionnaires are required to unveil them is a question for future research.

### Side effects

Although side effects such as seeing stars or tingling sensations have been describe for MRI, to present knowledge these side effects are not harmful. Accordingly, they are usually not considered in the clinical context. However, they may become relevant in the research setting, as they may constitute a factor of discomfort, for instance because participants may take them as indication of some harmful condition. We assessed such side effects by the question "Do you think you felt something strange caused by the MRI scanner?".

A surprisingly high number of 34% of the healthy subjects indeed noted something strange. However, a closer inspection of the comments revealed that 7 subjects just reported tiredness, which is well explained by the fact of lying still for 30–60 min and either doing nothing or working on a highly repetitive task. Five subjects reported dizziness during the scan, and two further subjects reported feeling slightly discoordinated and disoriented. Comments potentially relating to effects which would occur in the same context without the MRI scanner as well encompass notes such as (at the end of the scan) "seeing stars against the [bright] white screen", or "feeling wobbly when standing up after the end of the MRI scan" (i.e., after at least 40 min of lying motionless). Two subjects reported feeling slightly nauseated after a diffusion tensor imaging (DTI) scan, during which the scanner bed shakes noticeably. One participant reported a feeling of panic after some time, caused by the enclosed space in the MRI bore and the noise of the anatomical MPRAGE scan (however, the scan did not need to be aborted). Other comments, which cannot easily be categorized or explained comprised "heart rate fell into step with vibration of the scanner", "strange – during the last stage I saw 'things' flying about, but I couldn't focus on them", "Also I felt like I could feel something applied in the back of my head when the scan(s) started", "The sensation of magnets 'pulling' my head in various directions. Also my mind was making songs out of the noises", "isolated – like in a bubble, so all other perception strange", "it's hypnotic; felt like the bed was sinking downwards", "I felt a little bit hot (in my head too), but that's probably because I was tense". These latter comments may at least partially be caused by a heightened awareness or self-focused attention.

### Claustrophobia

Because participation was voluntary and participants have been warned about claustrophobia, we expected a lower rate of claustrophobia than the previously reported incidence of about 2% (e.g. [3,12]). Of the 70 healthy subjects, only one reported a feeling of claustrophobia (without the need to cancel the scan), which is an incidence of 1.4%. However, whether this 1.4% is significantly lower than the previously reported 2% cannot be answered reliably by the present data. Instead, we suggest that for reliable inferences with such low incidence rates much larger samples are required. Thus, future research is needed to test the hypothesis that a self-selection bias in the research setting results in a lower incidence of claustrophobia.

### Effect of gender

In healthy subjects, females rated the procedure significantly less comfortable than males. The reason for this difference is not clear, but may be found in the trend that women more often suffer from claustrophobia or panic attacks in the MRI environment [3,11,12,26]; and that they show increased levels of state anxiety before as well as after the MRI scan [6,8].

We observed the reversed pattern in patients: females found the procedure significantly more comfortable than males. This could be explained by the findings of Wollman et al. [4] who showed that, in the elderly, males find some aspects of the procedure less comfortable than females (such as lying flat, positioning, and not moving). However, further research is needed to decide whether this is a genuine gender by age interaction or whether these differences are due to some other differences in the imaging procedure.

### Effect of Age

Age did not affect any questionnaire item, which suggests that the perceived comfort is not affected by the age in the range of the investigated population (17 – 69 years). This is in line with Wollman et al. [4] who showed that two populations of elderly participants (means 76 and 92 years, respectively) did not differ significantly in their estimate of the overall comfort.

With respect to claustrophobia, it seems to be unclear whether age has an effect. While the findings of Eshed et al. [3] who showed that MRI-related claustrophobia is evenly distributed across all age groups between 20 and 80 years is in agreement with our findings, Sarji et al. [18] mentions non-significant age effects in their study of more than 3000 patients. In the so far largest study, Dewey et al. [12] found that in particular the age group 40 – 65 years is characterized by a higher incidence of claustrophobia (2.6%), while younger and older patients showed comparably lower rates of claustrophobia (1.3% and 1.5%, respectively). First, one should note that the incidence of claustrophobia is not necessarily related to the experienced comfort of the MRI scanning. Second, in our study age is confounded with the sample, i.e. the healthy subjects were younger (mean 26 years) than the patients (mean 54). Both groups did not only differ in age, but as well in other factors such as study duration which may result in opposite effects on the comfort as expected by the results of Dewey et al. [12]. Therefore, the discrepancy between the present results and Dewey et al. [12] may be due to the fact that claustrophobia and perceived comfort are largely unrelated, or to other differences in the study design.

### Effect of a DTI scan

The inclusion of a DTI scan had no effect. This is interesting, as the DTI sequence is characterized not only by a very different sounding gradient noise, but as well by a considerably shaking scanner bed. Thus, it seems that our findings are somewhat robust to changes in the employed MRI protocol.

### Study limitations

Approximately one third of the subjects and patients underwent an MRI scan before. Unfortunately, we do not have the data about the exact proportion, and we do not have the information which particular participants were scanned before, so that we cannot test for the effect of prior scanning on the perceived convenience. In the clinical context, MacKenzie et al. [6] assessed the previous imaging experience (MRI and computer tomography) and showed that on average previous experience did not affect the state anxiety before the MRI scan (compare also [21]). However, state anxiety before the MRI scan was lower if the previous experience was pleasant and higher when it was unpleasant. Assuming that in the basic research context participants only return if the experience has not been too unpleasant, one may speculate that these participants will most likely show on average more positive ratings in the present questionnaire as well. Thus, it may be that the perceived convenience is lower if a sample being scanned for the first time would be investigated. However, despite this potential bias we think that the present data are not unrepresentative of the everyday research fMRI scanning. The main reason for this is that most sites test participants repeatedly in different studies, and thus usually have a sample which consists of participants with prior scanning experience (of course, proportions may vary). Thus, we conclude that although the present data cannot disentangle the effect of prior scanning on perceived convenience, they are representative for the typical setting of basic fMRI research.

To our knowledge, no standardized questionnaire exists to assess general comfort during MRI scanning. Accordingly, we used a self-developed questionnaire for this purpose, as has been done by others [2,4,8]. However, to assess aspects related to comfort it probably is beneficial to use standardized questionnaires, such as the Claustrophobia Questionnaire (CLQ) [27] or the Spielberger State-Trait Anxiety Inventory.

An interesting option for future studies would be to use a pre- and a post-questionnaire, especially if it is known whether participants had prior experience with (functional) MRI. With such a procedure it would be possible to disentangle expectations about the MRI procedure from actual experience. In addition, it would be of interest whether different prior experiences (e.g. anatomical MRI of other body parts than head, anatomical MRI of head, functional MRI) result in the same (in)congruence between expectation and experience as assessed by a pre- and post-questionnaire. Such inferences cannot be made in the current study, since we assessed only the actual experience of the MRI scan, but not the prior expectations.

It should be noted that the present results are specific to our setting and MRI procedure and that there are a number of factors which may affect the scanning experience [12,22,28]. For instance, different variants of EPI sequences as well as different MRI machines can change the noise levels and characteristics. Furthermore, different scanner models may have different designs regarding depth and diameter of the bore, which may affect the feeling of claustrophobia. In the same way, different headcoils (e.g. narrow or closed ones), wearing goggles, or fixing the arms with straps or vacuum cushions may affect the comfort in general and the feeling of claustrophobia in particular.

## Conclusion

The present pilot study investigated the perceived comfort of the MRI procedure in the basic research setting. We showed that younger healthy subjects as well as elderly patients perceive the procedure as largely comfortable, without any major negative aspects. The reported level of comfort is at least comparable, if not even higher, as compared to clinical scans. This information can provide guidance to imaging researchers and ethical review boards.

## Competing interests

The authors declare that they have no competing interests.

## Authors' contributions

AJS led the study, developed the questionnaire, participated in data acquisition, and drafted the manuscript. SS participated in data acquisition and reviewed versions of the manuscript. AS was involved in the design of the study, interpretation of the data, and made revisions to draft versions of the manuscript.

## Pre-publication history

The pre-publication history for this paper can be accessed here:

http://www.biomedcentral.com/1471-2342/9/14/prepub

## References

[B1] Marshall J Martin T Downie J Malisza K A comprehensive analysis of MRI research risks: in support of full disclosure Can J Neurol Sci 2007 34 11 17 17352342 10.1017/s0317167100005734

[B2] Sparrow P Plein S Jones TR Thorley PJ Hale C Sivananthan MU Tolerance of MRI vs. SPECT myocardial perfusion studies – a patient survey J Magn Reson Imaging 2004 19 410 416 10.1002/jmri.20030 15065164

[B3] Eshed I Althoff CE Hamm B Hermann KG Claustrophobia and premature termination of magnetic resonance imaging examinations J Magn Reson Imaging 2007 26 401 404 10.1002/jmri.21012 17610281

[B4] Wollman DE Beeri MS Weinberger M Cheng H Silverman JM Prohovnik I Tolerance of MRI procedures by the oldest old Magn Reson Imaging 2004 22 1299 1304 10.1016/j.mri.2004.08.009 15607102

[B5] Thorp D Owens RG Whitehouse G Dewey ME Subjective experiences of magnetic resonance imaging Clin Radiol 1990 41 276 278 10.1016/S0009-9260(05)81665-4 2340700

[B6] MacKenzie R Sims C Owens RG Dixon AK Patients' perceptions of magnetic resonance imaging Clin Radiol 1995 50 137 143 10.1016/S0009-9260(05)83042-9 7889700

[B7] Flaherty JA Hoskinson K Emotional distress during magnetic resonance imaging N Engl J Med 1989 320 467 468 2913515 10.1056/nejm198902163200716

[B8] Dantendorfer K Amering M Bankier A Helbich T Prayer D Youssefzadeh S Alexandrowicz R Imhof H Katschnig H A study of the effects of patient anxiety, perceptions and equipment on motion artifacts in magnetic resonance imaging Magn Reson Imaging 1997 15 301 306 10.1016/S0730-725X(96)00385-2 9201677

[B9] Tornqvist E Mansson A Larsson EM Hallstrom I It's like being in another world – patients' lived experience of magnetic resonance imaging J Clin Nurs 2006 15 954 961 10.1111/j.1365-2702.2006.01499.x 16879539

[B10] Melendez JC McCrank E Anxiety-related reactions associated with magnetic resonance imaging examinations JAMA 1993 270 745 747 10.1001/jama.270.6.745 8336378

[B11] Katz RC Wilson L Frazer N Anxiety and its determinants in patients undergoing magnetic resonance imaging J Behav Ther Exp Psychiatry 1994 25 131 134 10.1016/0005-7916(94)90005-1 7983222

[B12] Dewey M Schink T Dewey CF Claustrophobia during magnetic resonance imaging: cohort study in over 55,000 patients J Magn Reson Imaging 2007 26 1322 1327 10.1002/jmri.21147 17969166

[B13] Kanal E Borgstede JP Barkovich AJ Bell C Bradley WG Felmlee JP Froelich JW Kaminski EM Keeler EK Lester JW American College of Radiology White Paper on MR Safety AJR Am J Roentgenol 2002 178 1335 1347 12034593 10.2214/ajr.178.6.1781335

[B14] Shen S Sterr A Szameitat A A template effect study on voxel-based morphometry in statistic parametric mapping Conf Proc IEEE Eng Med Biol Soc 2005 3 3051 3054 17282887 10.1109/IEMBS.2005.1617118

[B15] Shen S Szameitat AJ Sterr A VBM lesion detection depends on the normalization template: a study using simulated atrophy Magn Reson Imaging 2007 25 1385 1396 10.1016/j.mri.2007.03.025 17467945

[B16] Szameitat AJ Shen S Sterr A Motor imagery of complex everyday movements. An fMRI study NeuroImage 2007 34 702 713 10.1016/j.neuroimage.2006.09.033 17112742

[B17] Szameitat AJ Shen S Sterr A Effector-dependent activity in the left dorsal premotor cortex in motor imagery Eur J Neurosci 2007 26 3303 3308 10.1111/j.1460-9568.2007.05920.x 18005067

[B18] Sarji SA Abdullah BJ Kumar G Tan AH Narayanan P Failed magnetic resonance imaging examinations due to claustrophobia Australas Radiol 1998 42 293 295 10.1111/j.1440-1673.1998.tb00525.x 9833363

[B19] Tschirch FT Gopfert K Frohlich JM Brunner G Weishaupt D Low-dose intranasal versus oral midazolam for routine body MRI of claustrophobic patients Eur Radiol 2007 17 1403 1410 10.1007/s00330-006-0457-1 17093965

[B20] Tschirch FT Suter K Froehlich JM Studler U Nidecker A Eckhardt B Beranek-Chiu J Surber C Weishaupt D Multicenter trial: comparison of two different formulations and application systems of low-dose nasal midazolam for routine magnetic resonance imaging of claustrophobic patients J Magn Reson Imaging 2008 28 866 872 10.1002/jmri.21552 18821628

[B21] Thorpe S Salkovskis PM Dittner A Claustrophobia in MRI: the role of cognitions Magn Reson Imaging 2008 26 1081 1088 10.1016/j.mri.2008.01.022 18524527

[B22] Bangard C Paszek J Berg F Eyl G Kessler J Lackner K Gossmann A MR imaging of claustrophobic patients in an open 1.0T scanner: motion artifacts and patient acceptability compared with closed bore magnets Eur J Radiol 2007 64 152 157 10.1016/j.ejrad.2007.02.012 17374468

[B23] McGlynn FD Smitherman TA Hammel JC Lazarte AA Component fears of claustrophobia associated with mock magnetic resonance imaging J Anxiety Disord 2007 21 367 380 10.1016/j.janxdis.2006.06.003 16860972

[B24] Kilborn LC Labbe EE Magnetic resonance imaging scanning procedures: development of phobic response during scan and at one-month follow-up J Behav Med 1990 13 391 401 10.1007/BF00844886 2246785

[B25] Avrahami E Panic attacks during MR imaging: treatment with i.v. diazepam AJNR Am J Neuroradiol 1990 11 833 835 2114778 PMC8331638

[B26] Murphy KJ Brunberg JA Adult claustrophobia, anxiety and sedation in MRI Magn Reson Imaging 1997 15 51 54 10.1016/S0730-725X(96)00351-7 9084025

[B27] Radomsky AS Rachman S Thordarson DS McIsaac HK Teachman BA The Claustrophobia Questionnaire J Anxiety Disord 2001 15 287 297 10.1016/S0887-6185(01)00064-0 11474815

[B28] Heuck A Bonel H Huber A Muller-Lisse GU Sittek H Reiser M Patient acceptance of high-field whole-body MR systems, open MR-systems and dedicated MR systems for the extremities [German] Radiologe 1997 37 778 784 10.1007/s001170050282 9454270

